# Trends in the utilization of medicines sold in the private sector post- registration in South Africa and the implications for similar countries

**DOI:** 10.1186/s12889-023-15021-2

**Published:** 2023-01-28

**Authors:** Ntobeko Magnate Mpanza, Brian Godman, Mothobi Godfrey Keele, Moliehi Matlala

**Affiliations:** 1 Present Address: School of Pharmacy, Sefako Makgatho Health Sciences University, Pretoria, South Africa; 2 Strathclyde Institute of Pharmacy and Biomedical Sciences, University of Strathclyde, Glasgow, G4 0RE UK; 3 Centre of Medical and Bio-Allied Health Sciences Research (CMBHSR), Ajman University, Ajman, United Arab Emirates; 4 Department of Pharmacy and Pharmacology, University of the Witwatersrand, Johannesburgh, South Africa

**Keywords:** Dispensed medicines, Marketing authorisation, Private sector, Mental Health, Retail pharmacy, Multiple sourced medicines, Single Exit Price, South Africa, SAHPRA

## Abstract

**Background:**

Regulatory authorities register medicines for patients to access them within a reasonable period of time. There is a paucity of available data regarding the extent to which registered medicines reach the public after market authorisation is granted by the South African Health Products Regulatory Authority (SAHPRA). This is important since time spent by SAHPRA assessing medicines that are subsequently not launched onto the South African market means time wasted, which could be spent on assessing new medicines that address an unmet need in the country. Consequently, we initially analysed the time taken for registered medicines to reach patients and the relationship between medicines registered at SAHPRA and those subsequently dispensed in private pharmacies. The extent of registration of multiple sourced versus new patented medicines was also explored.

**Methods:**

A retrospective, descriptive and quantitative investigation was conducted for medicines registered between 2014 and 2019. Registered and dispensed medicines were compared to establish accessibility post registration. Data sources included SAHPRA and IQVIA datasets. Microsoft Excel and SAS were used for data storage, analysis, and computation of descriptive statistical analysis.

**Results:**

Of (*N* = 2175) registered medicines, only 358 (16.5%; 95% CI 15.0%—18.1%) were dispensed to patients, and out of 1735 medicines registered between 2015 and 2019, only 57 (3.3%; 95% CI 2.5%—4.2%) were dispensed during the study period. Medicines acting on the central nervous system were registered and dispensed the most at 21.0% and 18.0%, respectively, whereas antineoplastic and immunomodulation agents were registered and dispensed only 11% and 5%, respectively. A concern was that only 13.0% of registered medicines were originators, with most either as generics, including branded generics, or pseudo-generics.

**Conclusion:**

Regulatory measures should be implemented to ensure increased medicine access post-registration for new originators, especially for priority disease areas that benefit patients. Mental health diseases and improved access to oncology medicines require special attention and further investigation in South Africa.

## Background

Regulatory authorities allocate resources to evaluate the registration of pharmaceuticals to ensure the health and well-being of patients through assessing the efficacy, safety, and quality of new, re-formulated, and multiple-sourced medicines [1]. Similarly, manufacturers have a vested interest in the registration and sale of medicines within the shortest period of time. The latter is due to the fact that revenues can only be recouped through subsequent sales [2–5].

In South Africa, medicines are registered by the South African Health Products Regulatory Authority (SAHPRA) in terms of Sect. 15 of the Medicines and Related Substances Act 101 of 1965 (Medicines Act) [1, 5]. After marketing authorisation is granted, it is mandatory for manufacturers to enlist the Single Exit Price (SEP) of a registered medicine with the National Department of Health (NDoH) before such a medicine can be sold in the private sector [6, 7]. Schedule 0 medicines, e.g., paracetamol in small quantities, and veterinary medicines, e.g., florfenicol injection, are excluded from this price listing requirement. It is common for medicines to reach the market and be sold in the private sector, under certain conditions, without SAHPRA registration [8, 9]. This is allowed in terms of Sect. 21 of the Medicines Act, which makes provision for access to medicines on a named patient basis over a specified period [8, 10]. Sometimes these medicines eventually become registered. Generally, it takes no more than thirty working days for the NDoH to process a submission intended for listing the SEP [7]. Immediately after listing the SEP, nothing prevents the manufacturer from selling the medicine to retail outlets such as pharmacies where the public access medication.

The reasons for lack of immediate patient access to medicines after marketing authorisation may include the on-selling of the dossier of the registered medicine to another manufacturer, delays in approval of post-registration amendments to registration dossiers [3], and price erosion [11]. Price erosion occurs when the pharmaceutical company is forced to reduce the price of medicines for reimbursement below the price envisaged prior to registration. In this situation, the manufacturer may decide to terminate their plans to avoid revenue losses [3].This is mainly applicable to multiple sourced (generic) medicines, where companies must compete on prices for sustained sales.

Additional access delay factors also involve medical insurance schemes who either do not, or only partly reimburse, certain medicines, creating access problems for specific segments of the population [7, 12]. This results in patients having to pay out-of-pocket themselves for the shortfall to access partially reimbursed medicines. This partial reimbursement can lead to limited access to registered medicines, particularly among members who cannot afford the additional co-payment [2, 13].

The time lag taken to sell a medicine after registration is referred to as Time To Market (TTM) [12], with the “Patient W.A.I.T.” (Patients Waiting to Access Innovative Therapies) an indicator providing a benchmark to the rate of availability and waiting times for new medicines [12].

In Europe, patient access to new medicines is also highly variable. The average delay between market authorisation and patient access can vary by a factor greater than seven-fold [12]. The total time to patient access in Canada after regulatory approval and market authorisation also continues to increase [14]. In 2019, the average market access delay timelines were taken up by Health Technology Assessments (HTA) and pan-Canadian Pharmaceutical Alliance (pCPA) negotiation processes, which contributed 236 and 273 days, respectively [14]. In Bulgaria, marketing delay periods currently exceed 365 days despite efforts to reduce them [15].

In Africa, Southern African Development Community (SADC) countries are reported to experience delays in patient access to medicines [3]. However, the extent of this delay is currently not quantified.

Regulatory authorities especially in developing countries grapple with challenges in acquiring and retaining scarce and costly resources necessary to ensure expeditious registration of medicines [1, 3]. In view of this, the authorities must ensure these scarce resources are not misused and that the desired outcomes, which serve the broader health interests of the population, are achieved [5, 16]. Consequently, given the resources required by regulatory agencies to undertake their evaluations with typically more tasks than available personnel, it is reasonable to expect all registered medicines to be made available for sale and supplied to patients in the shortest possible time post-registration. However, the ultimate availability of new medicines will depend on various factors. These include potential reimbursement by the health authorities based on their proposed price, envisaged value, and likely budget impact [17, 18]. This is because there is increasing concern regarding the value and budgetary impact of new high-priced oncology medicines, or those to treat orphan diseases, which account for most new medicines being researched [19–21], and scarce resources to fund them especially in developing countries such as African countries [22, 23].

Due to currently limited availability of data on consumer access to medicines post marketing authorisation in South Africa, we sought to address this by determining the number and nature of registered medicines that are available in the private market in South Africa after market authorisation is granted by SAHPRA, as this is the first likely place of use. The study further aimed to establish the time it takes to start dispensing medicines in the private sector after registration. We believe a study of this nature is relevant because South Africa is in the process of implementing Universal Healthcare Coverage (UHC) policies, which seek to improve access to equitable healthcare [24], which is in line with Sustainable Development Goal 3 [25]. Consequently, the work performed by regulatory agencies in South Africa must be targeted to help ensure that the actual healthcare needs of the country’s population are met within the limited and scarce resources currently allocated to regulatory agencies in South Africa. As a result, resources should be prioritised where possible to assess the medicines that help address the current burden of disease rather than directed towards assessing medicines that are never launched exacerbated by price erosion. We are not sure this is currently the case, with applications for generics seeming to have a higher priority than new originator medicines which can improve the health of the population within acceptable costs [26]. This is a concern for the future.

Given this, the research questions are: Firstly, is there a relationship between registered and dispensed medicines and their Anatomical Therapeutic Chemical (ATC) classifications, which can identify the likely disease area for treatment? Secondly, what is the time typically taken to dispense medicines post registration at SAHPRA? Lastly, what proportion of multiple-sourced and originator medicines were registered in South Africa between 2014—2019? The findings can be used to suggest future priorities at SAHPRA to improve their efficiency and reduce time spent on appraising medicines that never reach the market. As a result, positively impacting the health of patients in South Africa and beyond.

## Methods

### Research design

The study investigated the impact of medicine registration outputs of the South African regulatory agency at a patient level. The study setting was the private sector spanning six years from 2014 to 2019. A retrospective, longitudinal, descriptive, and quantitative study was conducted using two datasets. This included data for medicines registered by SAHPRA between 2014 and 2019, and a different dataset for medicines dispensed between 2015 and 2019 provided by IQVIA. This is because dispensed data for the 2014 could not be obtained from IQVIA. However, given that a lag phase is expected between marketing authorisation and the availability of medicines at retail pharmacies, the cumulative account of dispensed data during the study period takes into account those medicines dispensed and available in the market in 2014. All analysed medicines were allocated into one of fourteen ATC classifications [27].

IQVIA collates data that is supplied to cash-paying and medically insured patients. The study period sought to represent the performance of the regulatory agency before, during, and after its transition in February 2018 from Medicines Control Council (MCC) to SAHPRA. The significance of medicines registered during the study period is that this time provides a good indicator of the maximum amount of time it can take for a medicine to remain un-dispensed in the South African private sector market post-registration**.** The private sector market was chosen for the analysis since medicines in the public sector are usually patent-expired; consequently, they are typically available as low-cost generics or biosimilars [20, 24, 28].

### Data sources

The data sources included the SAHPRA website for registered medicines [29] with IQVIA providing data for medicines dispensed in private sector pharmacies. Pharmacies are allowed to dispense any registered medicine regardless of whether it is a multiple-sourced or originator, including patent-protected medicines. They are not confined to dispensing medicines on the national tender, which is the case in the public and State facilities, including community health centres and hospitals [24]. The IQVIA dataset of dispensed medicines contained all the different pack sizes of a particular medicine, whereas the SAHPRA dataset represented the registered medicine without the different pack sizes. Consequently, the IQVIA dataset was larger than the SAHPRA dataset since the IQVIA dataset represented different pack sizes of the same medicine, which only appeared once in the SAHPRA dataset.

The medicines registered between 2014 and 2019 were categorised as originators, generics, or other. Others included pseudo generics, which the manufacturer of the originator medicine introduces just before patent expiry to extend sales, sometimes, as mentioned, referred to as ever-greening strategies [26, 30]. Branded generics are separate from the originator and multiple-sourced generic medicines (listed by their INN – International non-propriety name), for instance, in countries such as the United Kingdom [31]. This is different from the classification of Narsai et al. [1], who broke medicines down into just generics, new chemical entities, and vaccines, as we wanted to document the development of pseudo generics as this can add to costs with limited or no health gain [30]. ATC classifications were sourced from the World Health Organisation website [27].

### Sampling

Only those medicines registered between 2014 and 2019 and dispensed between 2015 and 2019 formed part of the study sample. A purposive sampling technique was adopted. The study period covered medicine registration outputs from the Medicines Control Council (MCC) and SAHPRA.

### Data collection and analysis

Quantitative data were collected and stored in MS excel spreadsheets prior to analysis. Microsoft Excel and SAS (SAS Institute Inc, Carey, NC, USA), Release 9.4.was used for the computation of descriptive statistical analysis in the form of percentages. During the comparison of registered and dispensed medicines, each registered medicine was compared to one or more pack sizes of the same medicine from the dataset of dispensed medicines. This served to verify the availability of the same medicine in both datasets. The analysis included all the different pack sizes and dosage forms of the same medicine dispensed in the retail pharmacy. This ensured that all formulations and pack sizes of the registered medicine were considered and accounted for during data analysis.

The expertise of a statistician was solicited to validate the study results. Microsoft Excel was used to compare the SAHPRA list of registered medicines and the IQVIA list of dispensed medicines to determine the time taken for each registered medicine to reach the marketplace. The year of registration and dispensing were the common variables in both the SAHPRA and IQVIA datasets; hence the period used in this study is described in years. Descriptive statistics were used, and data were presented as frequencies and percentages. The percentages assisted in ensuring that the proportions of the different datasets were compared. IQVIA provided the ATC classifications for dispensed medicines, which were verified using the WHO ATC classification dataset from the WHO website. The same ATC classification allocation strategy for medicines on the SAHPRA dataset was used.

### Validity and reliability

A pilot study was conducted to test the validity and appropriateness of the data collection tools. The pilot was deemed necessary especially given the large volumes of data available in systems where data required for the purposes of this study would be sourced. The pilot further sought to establish whether all the specific fields of information were available at the source. After completion, the data fields deemed unnecessary for the analysis were removed from the dataset. The officials responsible for data collation of registered and dispensed medicines at SAHPRA and IQVIA were subsequently requested to verify and provide inputs on the data collection tools.

## Results

### Medicines registered per year

A total of 2182 medicines were registered between 2014 and 2019, of which 2175 were analysed. Most medicines were registered in 2016 (484 /2175 = 22, 26%) with fewest medicines registered in 2018 (192/2175 = 8, 83%), In 2018 the authority transitioned from MCC to SAHPRA. However a slight increase in registrations was observed in 2019 (204/2175 = 9,38%) relative to 2018.

### The proportion of dispensed relative to registered medicines

**Fig. 1:**
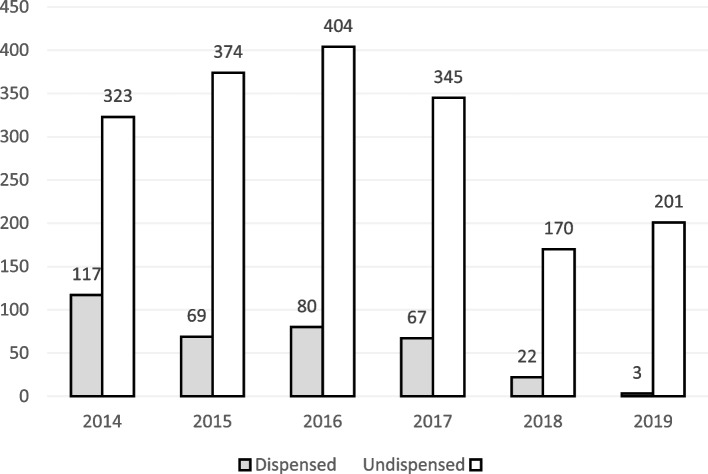
Proportion of dispensed and registered medicines

2014 had the highest percentage of dispensed medicines (117/358: 32.68%), and fewest medicines were dispensed in 2019 (3/358: 0.84%). Over the study period, the greatest number of dispensed medicines of those registered in the same year was in 2016 (404/484 = 83, 13%) (Fig. 1).

### Time taken to dispense medicines after registration

**Fig. 2:**
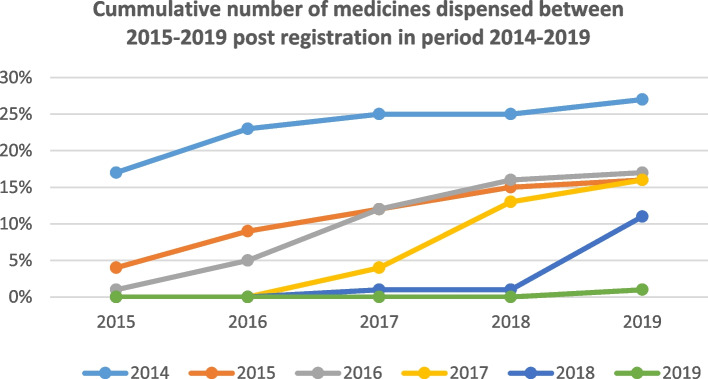
Time taken to dispense medicines post-registration in South Africa (2014–2019)

As seen in 2014, a maximum of 27% of registered medicines were dispensed within five years (Fig. 2). The highest percentage (24/484 = 5%) of medicines dispensed within the same year of registration was in 2016, whereas the smallest percentage (1%) of medicines dispensed within the same year of registration was in 2018 and 2019. Some medicines were dispensed before registration, as observed in 2016 and 2018, suggesting that some medicines tended to reach patients even before they were granted registration status at SAHPRA (Fig. 2).

### The relationship between ATC Classifications of registered and dispensed medicines in South Africa

Fourteen ATC Classifications for medicines registered between 2014 and 2019 and those dispensed between 2015 and 2019 were considered in the study, as shown in Table 1. The top three most registered medicines belonged to ATC Classifications Nervous System: ATC Code N (21%), Anti-infectives for systemic use: ATC Code J (20%), and the cardiovascular system: ATC Code C (16%). Similarly, the ATC Classifications of the top three most dispensed medicines acted on the nervous system (18%), anti-infectives for systemic use (18%), and the cardiovascular system (10%). The low utilisation of antiparasitic agents and anti-insecticides (280/20329 = 13,77%) is encouraging given the high burden of malaria infections in countries bordering South Africa.

**Table 1:** Alignment of ATC Classifications for registered and dispensed medicines in South Africa (2014–2019)

ATC CLASSIFICATION		REGISTERED MEDICINES RANKED AND CLASSIFIED INTO ATC CATEGORIES		DISPENSED MEDICINES RANKED AND CLASSIFIED INTO ATC CATEGORIES			
**CATEGORY**	**DESCRIPTION**	(N)	(%)	RANK	(N)	(%)	RANK
A	Alimentary Tract and Metabolism	137	6%	5	1992	10%	4
B	Blood and Blood forming organs	47	2%	9	1334	7%	7
**C**	Cardiovascular System	354	16%	3	2075	10%	3
D	Dermatologicals	35	2%	10	832	4%	10
G	GenitoUrinary System and Sex Hormones	101	5%	7	1087	5%	8
H	Systemic Hormonal Preparations	31	1%	11	200	1%	14
J	Anti-infectives for Systemic Use	444	20%	2	3666	18%	2
L	Antineoplastics and Immunomodulating Agents	245	11%	4	926	5%	9
M	Musculo Skeletal System	113	5%	6	1363	7%	6
N	Nervous System	465	21%	1	3687	18%	1
P	Antiparasitic products, insecticides, and Repellents	10	0%	14	280	1%	13
R	Respiratory System	81	4%	8	1914	9%	5
S	Sensory Organs	31	1%	11	414	2%	11
V	Various	21	1%	13	338	2%	12
	Other	61	3%		221	1%	
	**TOTAL**	**2175**	**100%**		**20,329**	**100%**	

General alignment existed in the ATC classifications of registered and dispensed medicines. However, antineoplastics and immunomodulating agents were found to be outliers, and medicines that fell into this ATC Classification group were registered more than they were dispensed.

### Registered medicines classified according to generic or originator status

Overall generic medicines accounted for 73.8% of the registered medicines, with new originators only accounting for 13.0% (Table 2).

**Table 2:** Proportion of originator and generic medicines registered in South Africa between 2014- 2019

Classification of medicines registered between 2014–2019		
Description	Total	%
Originator	283	13.0%
Generic	1604	73.8%
Other	288	13.2%
**Total**	**2175**	**100.0%**

The results showed that generics were registered most compared to originator medicines across the fourteen ATC classifications. This is not surprising considering that South Africa is pro-generic. The most registered originator medicines were antineoplastic and immunomodulating agents (16, 96%), followed by medicines acting on the cardiovascular system (16, 61%) and alimentary tract and metabolism (14,13%). The highest number of registered generics acted on the antiinfectives for systemic use (24,00%) and central nervous system (22, 19%) (Table 3).

**Table 3:** Generics and originator medicines registered in South Africa between 2014 -2019

		**Originator**		**Generic**		**Other**		**Total**	
**ATC classification**		**N**	**%**	**N**	**%**	**N**	**%**	**N**	**%**
A	Alimentary Tract and Metabolism	40	14.13%	76	4.74%	21	7.29%	137	6.30%
B	Blood and Blood forming organs	15	5.30%	21	1.31%	11	3.82%	47	2.16%
C	Cardiovascular System	47	16.61%	280	17.46%	26	9.03%	353	16.23%
D	Dermatologicals	11	3.89%	17	1.06%	7	2.43%	35	1.61%
G	Genito Urinary System and Sex Hormones	10	3.53%	88	5.49%	3	1.04%	101	4.64%
H	Systemic Hormonal Preparations	12	4.24%	9	0.56%	10	3.47%	31	1.43%
J	Anti-infectives for Systemic Use	33	11.66%	385	24.00%	26	9.03%	444	20.41%
L	Antineoplastic and Immunomodulating Agents	48	16.96%	163	10.16%	34	11.81%	245	11.26%
M	Musculo Skeletal System	12	4.24%	84	5.24%	17	5.90%	113	5.20%
N	Nervous system	29	10.25%	356	22.19%	80	27.78%	465	21.38%
P	Antiparasitic products, insecticides and	1	0.35%	8	0.50%	1	0.35%	10	0.46%
R	Respiratory System	16	5.65%	56	3,49%	9	3.13%	81	3.72%
S	Sensory Organs	0	0.00%	29	1.81%	2	0.69%	31	1.43%
V	Various	4	1.41%	14	0,87%	3	1.04%	21	0.97%
	other	5	1.77%	18	1.12%	38	13.19%	61	2.80%
**Total**		**283**	**13.0%**	**1604**	**8%**	**288**	**13.2%**	**2175**	**100.0%**

## Discussion

We believe this is the first study of this nature evaluating differences between the availability of medicines post registration in a developing country, especially one moving towards UHC. This builds on findings from the study of Narsai et al*.* and Vernaz et al. [1, 30]. We believe the results of this current study have important implications for countries such as South Africa, which has both a private and public healthcare system and where a single entity with limited resources is responsible for the registration of medicines for use in both sectors. The study has shown that there is currently a significant delay in patient access to medicines at community pharmacies.

Few medicines were dispensed within the same year of registration, with the majority of registered medicines remaining un-dispensed even after five years from the year of registration. Overall, a maximum of 27% of registered medicines can be expected to reach patients in private community pharmacies after five years from the year of registration. This is a significant concern as this wastes valuable regulatory resources.

Results further showed that a significantly large number of medicines are registered in South Africa relative to those which are dispensed. Potential reasons for this phenomenon could be low registration fees at SAHPRA. To address this, SAHPRA should consider increasing its fees particularly for medicines which have a predetermined number of existing competitors in the market. However, this has to be balanced against the potential benefits of increased competition lowering prices as seen in the Netherlands where competition resulted in the prices of generics at just 2% of pre-patent loss prices [32]. Lower fees could however be considered for medicines which are deemed crucial to address unmet medical needs and those identified as essential medicines and for treatment of rare diseases.

Similar to the findings of Narsai et al*.* [1] in South Africa, who found 90.8% of medicines registered between 2012 and 2017 were generics, we also found that the majority of medicines registered during our study period were generics. These results are also similar to those in the Norwegian setting, where many generic medicines which were granted Market Authorisation by the Norwegian Medicine Agency (NoMA) only entered the market after a while [33]. This time lag is compounded by many medicines registered in 2014 in South Africa remaining un-dispensed in the private sector by 2019.

A key question remains in South Africa: What has happened to the registered medicines that were never dispensed in community pharmacies? Given that manufacturers can supply registered medicines either in the public or private sector or both, it is possible that some of the missing medicines were registered for supply to the public sector. Alternatively, specific dynamics along the supply chain could contribute to the lack of availability and dispensing of certain medicines among community private sector pharmacies. At the manufacturer's level, for example, potential reasons may relate to changes in the conditions of the market between the time of submitting the medicine registration dossier and the timing of registration of those particular medicines [33]. If the manufacturer experiences circumstances such as price erosion, which are deemed to render the registered medicines unviable to sell, it is unlikely such a medicine will be brought into the market and therefore dispensed. According to Narsai et al. [3], manufacturers also terminate the supply of their registered medicines because of registration renewal and Good Manufacturing (GMP) inspection fees, earlier introduction into the market of a more innovative or convenient dosage form, delays in approval of post-registration amendments to registration dossiers and the availability of cheaper generics. In addition, stringent country-specific labelling requirements can cause pharmaceutical companies to resolve not to supply medicines in certain markets.

Encouragingly, medicines acting on the nervous and cardiovascular systems and anti-infective medicines for systemic use were registered and dispensed the most in the present study. This is in line with the current burden of disease patterns in South Africa, except for the high utilisation of mental health medicines that act on the nervous system which may not be receiving the attention they deserve [34]. We have seen this across Africa, where there are concerns with the management of patients with mental health disorders. This situation is exacerbated by the limited number of trained professionals across Africa [35], made worse by the COVID-19 pandemic [36, 37].

The antineoplastic and immunomodulating agents were the most registered originator medicines, which raises concerns about the increasing burden of non-communicable diseases in South Africa [1, 38]. There is also concern that most registered generics, the largest group of registered medicines, were anti-infectives for systemic use, which includes antibiotics and tuberculosis treatment. Treatments for tuberculosis (TB) are expected to be dispensed the most since TB is currently recorded as the leading cause of most deaths in South Africa [38, 39]. The high utilisation of antibiotics and the subsequent implications for continued antimicrobial resistance (AMR) development in South Africa are of concern, and this also needs addressing, building on the national AMR strategy [39, 40].

Encouragingly, the results also showed general alignment of registered medicines and those dispensed among private sector retail pharmacies in South Africa in terms of their ATC classification (Table 1). The only outlier was oncology medicines. This is not surprising as most countries are grappling with the growing number of new premium price cancer therapies, often with limited health gain [41–43]. This raises issues regarding the ability to reconcile access to costly oncology treatments and efficient spending to ensure the sustainability of healthcare systems [41, 44], with proposals from health authorities and governments to improve the pricing of new cancer medicines [44]. The generally high launch price of new oncology medicines could be a considerable contributor to the lack of dispensing of these medicines. However, the prices of a number of standard oncology treatments are decreasing with the increasing availability of lower-cost oral generics and biosimilars [42, 44–46]. We are also aware of considerable price differences for oncology medicines in the private sector in South Africa, which may further impact the availability of certain brands even though they are licensed [47]. The increasing unaffordability of oncology medicines could contribute to their lower than expected utilisation in the private sector. This is despite the registration of these medicines. In addition, the time taken to include oncology medicines on formulary lists of medical schemes or accept them for reimbursement could contribute to their delayed utilisation.

According to the World Health Organisation (WHO), when manufacturers price medicines solely according to commercial goals, a health system’s ability to achieve public health goals is impaired because high prices limit patient access, thereby limiting the full potential of the innovation [48]. With the number of people living with cancer predicted to increase in the coming decades, the unaffordability caused by the high prices of new cancer medicines will only worsen if the current pricing trend continues [44, 48]. In addition, several antineoplastic and immuno-modulating agents are likely to be only administered in the hospital, which would be excluded from our analysis given the study design. The is considered one of the limitations of this study. Similarly, medicines that could not be found on the list of medicines dispensed in retail pharmacies include a large volume of parenteral formulations, with injectables again not typically dispensed in retail pharmacies. It should also be noted that the large number of dispensed medicines could be because certain medicines and their various pack sizes were dispensed more than once within the same year and over the study period. This is particularly relevant to medicines used to treat chronic diseases and those in demand. Nonetheless, the data presented in this study is helpful in understanding the proportional differences between registered and dispensed medicines.

We believe the results from this study can contribute to knowledge development to improve regulatory processes and ensure efficiencies in the regulatory system. According to Bujar et al. [49], it is crucial to continually improve the internal decision-making practices of regulatory authorities to ensure quality is built into the process and to guarantee that accurate information from the past is available to inform current and future decisions. In addition, available resources are used as optimally as possible.

As mentioned, a high percentage of generics (73%) registered during the study period is similar to the findings of Narsai et al*.* [1]. The 13% of medicines that include pseudo generics and branded generics require further scrutiny. These figures indicate levels of ever-greening and pseudo-generics introduced by manufacturers of innovative medicines to help extend their franchise sales [26, 30]. While pseudogenerics might benefit the manufacturer, they do not necessarily provide long term financial benefits for the patient. According to Bangalee and Suleman [26], the price difference between pseudo-generic and authentic generic medicines can be up to 40%. Despite this, pseudo-generics maintain market domination, as patent/originator medicine manufacturers have a first-mover or market entry advantage, which impedes natural competition and ultimately increases medicine prices [26]. Given the continued increase in overall healthcare costs, every effort should be made to educate patients about differences in the types of medicines on offer, empowering them to make informed purchasing decisions.

Overall, the results suggest potential limitations for the entry of originators and innovative interventions into the South African health market. This needs to be addressed going forward to benefit all key stakeholder groups, most notably to adequately address the country's burden of disease. This study points to the need for regulators to triangulate datasets to establish the disease areas of prime importance in South Africa. Utilising a limited set of approaches in determining public health focus areas for South Africa could lead to the erroneous exclusion of certain diseases during priority setting by regulators. This needs to be addressed going forward.

Despite the unavailability of an independent and separate dataset for medicines dispensed in 2014, we are confident about the quality of results and their reliability to draw pertinent conclusions. The study duration of six years and the analysis of all and not some dispensed medicines is deemed adequate to address any biases potentially caused by missing data.

## Conclusion

The high number of medicines that remain inaccessible to consumers after regulatory authorities have allocated resources towards ensuring medicine registration is of great concern and requires additional scrutiny.

Access to originator medicines should be improved and balanced against the appreciable number of generics that are registered but never launched. In other settings, regulatory authorities use the "sunset clause" to encourage manufacturers to market medicines within a specific time post registration and to avoid the withdrawal of registration status by the regulatory authorities [50]. The Heads of the Medicines Agency (HMA) compile and publicly share information on all medicines that are unavailable aftermarket authorization is granted [51]. Such approaches could be considered for South Africa.

SAHPRA could also consider differential pricing based on unmet need for new medicines as well as collaborate with pharmaceutical sector associations to ensure that all registered medicines are accessible as far as possible post-market authorisation.

## Data Availability

Registered medicines were retrieved from the SAHPRA website HTTPS://www.sahpra.org.za/registered-health-products.The ATC classifications of all the medicines included in the analysis were found on the WHO website https://www.who.int/tools/atc-ddd-toolkit/atc-classification. IQVIA supplied the list of medicines dispensed in the private sector pharmacies used in the analysis.

## Abbreviations

AMR: Antimicrobial Resistance
ATC: Anatomical Therapeutic Chemical
EFPIA: European Federation of Pharmaceutical Industries and Associations
GMP: Good Manufacturing Practice
HTA: Health Technology Assessment
MCC: Medicines Control Council
NDoH: National Department of Health of South Africa
NoMA: Norwegian Medicine Agency
pCPA: pan-Canadian Pharmaceutical Alliance
SADC: Southern African Development Community
SAHPRA: South African Health Products Regulatory Authority
SEP: Single Exit Price
TTM: Time To Market
UHC: Universal Health Coverage
WHO: World Health Organisation

## References

[1] Narsai K, Leufkens, H G M and Mantel‑Teeuwisse A K . Linking market authorizations of medicines with disease burden in South Africa. Journal of Pharmaceutical Policy and Practice. 2021. Available on https://joppp.biomedcentral.com/track/pdf/10.1186/s40545-021-00314-x.pdf. 10.1186/s40545-021-00314-x PMC8017838 33795015

[2] Kanavos P, MacLehose L, McKee M. Border-crossing patients GATS and health services Fair trade: health and safety Movement of health professionals Beyond the candidate countries: health and the stability pact countries. Gateway to the European Union: health and EU enlargement. Euro health, (Autumn 2002) Volume8 Number 4, Special Issue, Autumn 2002 ISSN1356–1030. Euro Health. Available from: https://www.lse.ac.uk/lse-health/assets/documents/eurohealth/issues/eurohealth-v8n4.pdf#page=28.

[3] Narsai K, Williams A, Mantel-Teeuwisse AK (2012). Impact of regulatory requirements on medicine registration in African countries – perceptions and experiences of pharmaceutical companies in South Africa. Southern Med Review.

[4] Prašnikar J, Škerlj T (2006). New product development process and time-to-market in the generic pharmaceutical industry. Ind Mark Manag.

[5] Sithole T, Mahlangu G, Salek S, Walker S (2020). Evaluating the Success of ZaZiBoNa, the Southern African Development Community Collaborative Medicines Registration Initiative. Ther Innov Regul Sci.

[6] MCC. Registration of Medicines. General Information. Available from http://www.kznhealth.gov.za/research/mccinfo.pdf.20087.

[7] Mpanza N. Factors that influence medical scheme insured consumers to co-pay for prescription medicines at private community pharmacies in Pretoria, Gauteng Province, South Africa. 2016. Available from https://etd.uwc.ac.za/handle/11394/5477.

[8] SAHARA. Section 21 Access to Unregistered Medicines September 20. 2020. Available from https://www.sahpra.org.za/wp-content/uploads/2020/12/2.52_Section_21_Access_to_Unregistered_Medicines_Sept20_v2-003.pdf.

[9] Mpanza NM, Bradley H, Laing R (2019). Reasons why insured consumers co-pay for medicines at retail pharmacies in Pretoria, South Africa. Afr J Prm Health Care Fam Med.

[10] MCC. Section 21 application. Council's responsibilities and liability when performing its function in terms of section 21 of ACT 101 OF 1965. Available from http://www.denovomedica.com/cpd-online/wp-content/uploads/Section-21-Application.pdf.

[11] Gursoy, K. How is current pharmaceuticals pricing policy on Generics performing in Turkey regarding price erosion? DOI: 10.21441/sguz. 2017.5 Available from https://www.researchgate.net/publication/315921063_how_is_current_pharmaceuticals_pricing_policy_on_generics_performing_in_Turkey_regarding_price_erosion. 10.1016/j.jval.2014.08.948 27200992

[12] Machin C. (2018). EFPIA Market Access Delays Analysis. Available from http://www.hull.hr/wp-content/uploads/2018/04/Market-Access-Delays-2017-Final-140318-1.pdf.

[13] Discovery. Discovery Health medical scheme oncology programme.2021. Available from https://www.discovery.co.za/wcm/discoverycoza/assets/medical-aid/benefit-information/2021/oncology-programme-2021.pdf.

[14] Salek S, Hoskyn SL, Johns JR, Allen N, Sehgal C (2019). Factors influencing delays in patient access to new medicines in canada: a retrospective study of reimbursement processes in public drug plans. Front Pharmacol.

[15] Tsekov I, Dimitrova M, Voynikov Y. Role of the EMA specific marketing authorization procedures for early access on the time to patient access in Bulgaria. 2021.

[16] Ndomondo-Sigonda M, Miot J, Naidoo S, Masota NE, Ng’andu B, Ngum N, and Kaale E. Harmonization of medical products regulation: a key factor for improving regulatory capacity in the East African Community. 2021. Available from: DOI10.1186/s12889-021-10169-1. 10.1186/s12889-021-10169-1 PMC7818747 33478421

[17] Eriksson I, Wettermark B, Persson M, Edström M, Godman B, Lindhé A, Malmström RE, Ramström H, von Euler M, Bergkvist Christensen A. The Early Awareness and Alert System in Sweden: History and Current Status. Front Pharmacol. 2017;8:674 Available from: Frontiers in Pharmacology. 2017 Oct 5;8:674. DOI: 10.3389/fphar.2017.00674. eCollection 2017. PMID: 29056910. 10.3389/fphar.2017.00674 PMC5635816 29056910

[18] Godman B, Bucsics A, Vella Bonanno P, Oortwijn W, Rothe CC, Ferrario A, et al. Barriers for Access to New Medicines: Searching for the Balance Between Rising Costs and Limited Budgets. Front Public Health. 2018;6:328; Frontiers in Public Health. 2018 Dec 5;6:328. DOI: 10.3389/fpubh.2018.00328. eCollection 2018.PMID: 3056893. 10.3389/fpubh.2018.00328 PMC6290038 30568938

[19] Luzzatto L, Hyry HI, Schieppati A, Costa E, Simoens S, Schaefer F (2018). Outrageous prices of orphan drugs: a call for collaboration. Lancet.

[20] Godman B, Leong T, Abubakar AR, Kurdi A, Kalemeera F, Rwegerera GM (2021). Availability and Use of Long-Acting Insulin Analogues Including Their Biosimilars across Africa: Findings and Implications. Intern Med.

[21] IQVIA. Global Trends in R&D OVERVIEW THROUGH 2021. 2022. Available at: https://www.iqvia.com/-/media/iqvia/pdfs/institute-reports/global-trends-in-r-and-d-2022/iqvia-institute-global-trends-in-randd-to-2021.pdf?_=1648547404724.

[22] Baumgart DC, Misery L, Naeyaert S, Taylor PC (2019). Biological Therapies in Immune-Mediated Inflammatory Diseases: Can Biosimilars Reduce Access Inequities?. Front Pharmacol.

[23] Al-Ziftawi NH, Shafie AA, Mohamed Ibrahim MI (2021). Cost-effectiveness analyses of breast cancer medications use in developing countries: a systematic review. Expert Rev Pharmacoecon Outcomes Res.

[24] Meyer JC, Schellack N, Stokes J, Lancaster R, Zeeman H, Defty D (2017). Ongoing Initiatives to Improve the Quality and Efficiency of Medicine Use within the Public Healthcare System in South Africa Preliminary Study. Front Pharmacol.

[25] GBD 2019. United Nations (2020). Measuring universal health coverage based on an index of effective coverage of health services in 204 countries and territories, 1990-2019: a systematic analysis for the Global Burden of Disease Study 2019. Lancet.

[26] Bangalee V, Suleman F. Pseudo-Generics in South Africa: A Price Comparison. 2019. Available from: https://www.ncbi.nlm.nih.gov/pmc/articles/PMC6754781/. DOI: 10.1016/j.vhri.2019.06.001 10.1016/j.vhri.2019.06.001 PMC6754781 31357098

[27] WHO. Anatomical Therapeutic Chemical (ATC) Classification. 2021. Available at: https://www.who.int/tools/atc-ddd-toolkit/atc-classification.

[28] Gray A, Suleman F, Pharasi B. South Africa’s National Drug Policy: 20 years and still going. South African Health Review. 2017; 1 : 49–58. Available at: https://www.hst.org.za/publications/South%20African%20Health%20Reviews/HST%20SAHR%202017%20Web%20Version.pdf

[29] SAHPRA (2021). Registered health products. Available from: HTTPS:// www.sahpra.org.za/registered-health-products.

[30] Vernaz N, Haller G, Girardin F, Huttner B, Combescure C, Dayer P (2013). Patented drug extension strategies on healthcare spending: a cost-evaluation analysis. PLoS Med.

[31] S MacBride-Stewart S McTaggart A Kurdi J Sneddon S McBurney B Godman do Nascimento RCRM et al. Godman B 2021 Initiatives and reforms across Scotland in recent years to improve prescribing; findings and global implications of drug prescriptions Int J ClinExp Med. 14 12 2563 86

[32] Woerkom M, Piepenbrink H, Godman B, Metz J, Campbell S, Bennie M (2012). Ongoing measures to enhance the efficiency of prescribing of proton pump inhibitors and statins in The Netherlands: influence and future implications. J Comp Eff Res.

[33] Kabashi V. Generic competition in the pharmaceutical industry - How long does it take for generic drugs to enter the market after having applied for Market Authorization in Norway? 2013. Available from https://www.duo.uio.no/handle/10852/38419.

[34] Modjadji P. Communicable and non-communicable diseases coexisting in South Africa. The Lancet, Global Health. Published: July 2021 Available from::10.1016/S2214-109X(21)00271-0. 10.1016/S2214-109X(21)00271-0 34143983

[35] Godman B, Grobler C, Van-De-Lisle M, Wale J, Barbosa WB, Massele A (2019). Pharmacotherapeutic interventions for bipolar disorder type II: addressing multiple symptoms and approaches with a particular emphasis on strategies in lower and middle-income countries. Expert Opin Pharmacother.

[36] Kar, S K, Arafat, SMY, Kabir, R, Sharma, P, and Saxena, SK. Coping with Mental Health Challenges During COVID-19. Coronavirus Disease 2019 (COVID-19). 2020 : 199–213. doi: 10.1007/978-981-15-4814-7_16. PMCID: PMC7189395.

[37] Chen J, Farah N, Dong RK, Chen RZ, Xu W, Yin J (2021). Mental Health during the COVID-19 Crisis in Africa: A Systematic Review and Meta-Analysis. Int J Environ Res Public Health.

[38] Stats SA. Mortality and causes of death in South Africa: Findings from death notification 2018. 2021. Available from: http://www.statssa.gov.za/publications/P03093/P030932018.pdf.

[39] Godman B, Egwuenu A, Wesangula E, Schellack N, Kalungia AC, Tiroyakgosi C, et al. Tackling antimicrobial resistance across sub-Saharan Africa; current challenges and implications for the future. Expert Opinion on Drug Safety. 2022 10.1080/14740338.2022.2106368 35876080

[40] Engler D, Meyer JC, Schellack N, Kurdi A, Godman B (2021). Compliance with South Africa's Antimicrobial Resistance National Strategy Framework: are we there yet?. J Chemother.

[41] Cohen D (2017). Cancer drugs: high price, uncertain value. BMJ.

[42] Godman B, Hill A, Simoens S, Kurdi A, Gulbinovič J, Martin AP, Timoney A (2019). Pricing of oral generic cancer medicines in 25 European countries; findings and implications. Gener Biosimilars Initiative J.

[43] OECD. Addressing Challenges in Access to Oncology Medicines Analytical Report. 2020. Available from: https://www.oecd.org/health/health-systems/Addressing-Challenges-in-Access-to-Oncology-Medicines-Analytical-Report.pdf. 2020.

[44] Godman B, Hill A, Simoens S, Selke G, Selke Krulichová I, Zampirolli Dias C (2021). Potential approaches for the pricing of cancer medicines across Europe to enhance the sustainability of healthcare systems and the implications. Expert Rev Pharmacoecon Outcomes Res.

[45] Yang J, Yu S, Yang Z, Yan Y, Chen Y, Zeng H (2019). Efficacy and Safety of Anti-cancer Biosimilars Compared to Reference Biologics in Oncology: A Systematic Review and Meta-Analysis of Randomized Controlled Trials. BioDrugs.

[46] Jang M, Simoens S, Kwon T (2021). Budget Impact Analysis of the Introduction of Rituximab and Trastuzumab Intravenous Biosimilars to EU-5 Markets. BioDrugs.

[47] Mattila PO, Babar ZU, Suleman F (2021). Assessing the prices and affordability of oncology medicines for three common cancers within the private sector of South Africa. BMC Health Serv Res.

[48] WHO. Access to medicines, vaccines, and pharmaceuticals technical report pricing of cancer medicines and its impacts. Available from https://apps.who.int/iris/bitstream/handle/10665/277190 9789241515115-eng.pdf (who. int).

[49] Bujar M, McAuslane N, Connelly P, Walker SR. Quality Decision‑Making Practices in Pharmaceutical Companies and Regulatory Authorities: Current and Proposed Approaches to Its Documentation. 2020. Available from https://link.springer.com/content/pdf/10.1007/s43441-020-00167-7.pdf. 10.1007/s43441-020-00167-7 PMC7704497 32472442

[50] EMA. European Medicines Agency Post-authorisation Evaluation of Medicines for Human Use. 2006. Available from: https://www.ema.europa.eu/en/documents/scientific-guideline/guideline-assessment-genotoxicity-herbal-substances/preparations_en.pdf.

[51] HMA. Availability of medicines for human use. Availability problems of medicinal products for human use. 2021.

